# Tumor-induced osteomalacia: a case report

**DOI:** 10.1186/s13256-021-03220-7

**Published:** 2022-01-12

**Authors:** Khalid Aligail, Joel A. Dave, Ian Louis Ross

**Affiliations:** Division of Endocrinology, Department of Medicine, Faculty of Health Sciences, Groote Schuur Hospital and University of Cape Town, J47-85 Old Main Building, Private Bag X3, Observatory, Cape Town, 7935 South Africa

**Keywords:** Tumor-induced osteomalacia, Osteomalacia, Hypophosphatemia, Fibroblast growth factor-23

## Abstract

**Background:**

Tumor-induced osteomalacia is a rare, acquired paraneoplastic syndrome, including hypophosphatemia, high serum alkaline phosphatase, reduced active vitamin D, suboptimal bone mineral density, bone pain, fragility fractures, and muscle weakness.

**Case presentation:**

We report a case of 74–year–old male of mixed ancestry with hypophosphatemia resistant to treatment despite optimal compliance, associated with profound reduction of bone mineral density and multiple nontraumatic fractures, including bilateral rib fractures, lower-thoracic (T11, T12) vertebrae, and two fractures involving the surgical and anatomical neck of the right humerus. We discuss an approach to identifying the underlying cause of hypophosphatemia associated with fragility fractures, and options for management of this rare condition.

**Conclusion:**

Although rare, tumor-induced osteomalacia can be diagnosed if a logical stepwise approach is implemented. Surgery could be curative if the tumor is properly located and is resectable.

## Background

Osteomalacia usually manifests with a predictable pattern of biochemical derangements, including hypophosphatemia, elevated serum alkaline phosphatase, and low or inappropriately normal active vitamin D, associated with reduced bone mineral density [1]. Additional clinical manifestations of osteomalacia include bone pain, fragility fractures, and muscle weakness, and in children, rickets may lead to severe stunting of their longitudinal growth, if not managed optimally [2–4]. Several disorders resulting in hypophosphatemia can cause osteomalacia by directly limiting the mineralization process [5, 6]. Vitamin D deficiency, resulting in malabsorption, for example from celiac disease, defective hydroxylation of vitamin D, or resistance to vitamin D, may result in deficient plasma calcium and phosphate concentrations, and in the long term, can cause osteomalacia [7, 8]. Phosphate deficiency may also result from poor dietary intake or renal losses associated with Fanconi syndrome, hypophosphatemic osteomalacia, and in tumor-induced osteomalacia (TIO) [1, 5]. The pathogenic mechanism of TIO is the excessive secretion of fibroblast growth factor 23 (FGF-23), a phosphatonin secreted by the tumor arising from mesenchymal tissue, which can result in reduced renal phosphate reabsorption and impaired vitamin D 1α-hydroxylase activity [1, 9].

Laboratory workup to elucidate the cause of hypophosphatemia includes confirmation of reduced renal tubular reabsorption of phosphate, serum phosphate and calcium, alkaline phosphatase, 25-hydroxy vitamin D, 1–25-hydroxy vitamin D, genetic testing for hypophosphatemic osteomalacia, and, if available, FGF-23 concentrations [3, 4]. Treatment includes the combination of vitamin D (calcitriol and alfacalcidol) and phosphate supplementation. If a proven mesenchymal tumor is identified consistent with TIO, surgical resection is recommended and is potentially curative [2, 9, 10].

## Case presentation

A 74-year–old man of mixed ancestry, married father and pensioner, with neither significant past medical history nor a relevant family history, was referred to the endocrine service because of severe osteoporosis. The diagnosis of osteoporosis was made based on the findings of a dual x-ray absorptiometry (DEXA) scan, performed by a private radiology practice, which showed severely low bone mineral density, with T-scores of the lumbar spine (L1–L4) of −3.1 standard deviations (SD), left femoral neck −4.3 SD, left total hip −3.4 SD, right femoral neck −4.7 SD, right total hip −4.1 SD, and right forearm −1.3 SD.

His history is noteworthy for long-standing generalized body pain and muscle weakness for about 7 years. His muscle weakness progressed in severity rendering him bed bound for 2 years prior to presentation. At the time of his review by the endocrine service, he was only taking calciferol and calcium supplementation but did not demonstrate any improvement in mobility. Significant proximal muscle weakness (4/5) was elicited at both the shoulder and pelvic girdles, with generalized muscle wasting. His family observed progressive loss of longitudinal height and noted that he tired easily. There were no apparent symptoms suggestive of an occult malignancy. He had neither a suggestive family history of hypophosphatemic osteomalacia, nor Fanconi syndrome. Moreover, he was a life-long nonsmoker and consumed no alcohol.

Additional clinical examination revealed a bed-bound elderly man, with a barrel chest, and a flail segment of his chest, consistent with rib fractures. Hemodynamically, he was stable, blood pressure was 130/85 mmHg and pulse rate 82 per minute in sinus rhythm. He used accessory respiratory muscles in keeping with respiratory distress. Chest auscultation revealed bilateral scattered crackles. Importantly, he was severely kyphotic, and the rest of the systemic examination was noncontributory.

Initial laboratory workup showed that his serum creatinine was 59 (64–104) µmol/L, full blood count, liver function tests, thyroid function tests, uric acid, and lipogram were all within normal limits. Moreover, additional tests revealed a serum phosphate of 0.39 (0.78–1.4) mmol/L, calcium 2.01 (2.2–2.56) mmol/L, albumin 46 (35–52) g/L, alkaline phosphatase 302 (53–128) U/L, and a simultaneous measure of parathyroid hormone of 16.8 (1.6–6.9) pmol/L. We confirmed that he was deficient in serum 25–hydroxy-vitamin D [45.2 (replete > 72.5) nmol/L]. His fractional excretion of phosphate was 29% (< 5%) and the ratio of tubular maximum reabsorption of phosphate to glomerular filtration rate (TmP/GFR) was 0.33 mmol/L (normal range 0.8–1.35 mmol/L), taken contemporaneously with a serum phosphate of 0.44 (0.78–1.4) mmol/L, indicates profound phosphaturia. Venous blood gas showed a pH of 7.38 (7.35–7.45), pCO2 5.6 (4.66–6.38) kPa, HCO3 25 (19–24) mmol/L, and the erythrocyte sedimentation rate was 4 (0–20) mm/hour. Additional tests to identify the underlying cause for his fragility fractures included a normal serum protein electrophoresis, absence of Bence–Jones proteins, and an 08:00 serum testosterone of 13.4 (6.7–25.7) nmol/L. Antitissue transglutaminase antibodies, antigliadin, and endomysial antibodies were additionally negative. In view of his particularly high phosphaturia, exclusion of Fanconi syndrome was undertaken, which demonstrated normal urinary excretion of amino acids, absence of glycosuria, and sodium and potassium excretion in the urine were entirely within normal limits.

**Fig. 1:**
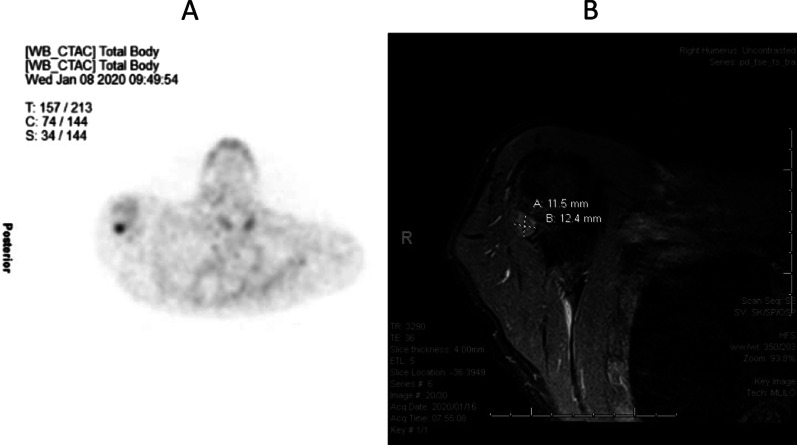
Demonstration of a tumor in the right humeral head by Gallium 68-DOTANOC PET/CT (**A**) and magnetic resonance imaging (MRI) (**B**)

He was continued on calciferol and alfacalcidol and commenced on phosphate supplementation. Despite regular use of these medicines and normalization of his serum calcium and vitamin D levels, low serum phosphate and elevated alkaline phosphatase and parathyroid hormone persisted. Oncogenic osteomalacia was considered having excluded a genetic form of hypophosphatemic osteomalacia. A Gallium Ga 68-DOTANOC PET/CT showed a focus of avid uptake in the soft tissue of the posterior aspect of the right humeral head, and a representative magnetic resonance imaging (MRI) scan showed an ill-defined nodular lesion corresponding with the initial area of uptake, measuring 12 × 12 mm, related to the distal teres minor muscle fibers (Fig. 1). Associated with the defined lesion on MRI scan, two fractures of the right head of humerus were seen, with one of the fractures being recently sustained. Multiple rib fractures were also seen. The lesion in the right shoulder was considered to be the likely culprit. Due to our resource limitations, it was deemed too costly to initiate somatostatin analog therapy, and surgery was postponed until the end of the COVID-19 pandemic.

## Discussion

We present a rare case of hypophosphatemic osteomalacia, resulting in severe osteoporosis and multiple fractures from tumor-inducing osteomalacia complicated by severe vitamin D deficiency. Although we did not have histological evidence of a mesenchymal tumor, we feel that there is sufficient indirect evidence for this, based on the positive Gallium Ga 68-DOTANOC PET/CT results and confirmation of a well-defined lesion on MRI.

Severe reduction in bone mineral density and fragility fractures can, rarely, be caused by hypophosphatemia [11]. Specifically, tumor-induced osteomalacia (TIO) is a rare, frequently missed condition, which is challenging to diagnose. The symptoms and biochemical derangements are often vague and mirror other conditions, for example, osteoporosis. It is important to know that inadvertent use of bisphosphonates could have aggravated the condition considerably [12]. Until 2018, only 500 cases were reported worldwide [13], with a peak age distribution of between 40 and 45 years [14].

TIO is a rarely acquired, paraneoplastic syndrome, resulting in hypophosphatemia, low levels of active vitamin D, severe osteomalacia, bone pain, fragility fractures, and muscle weakness. Patients with TIO are generally symptomatic for bone pain, muscle weakness, and gait disturbance, likely a consequence of the pelvic girdle myopathy [2]. A Chinese retrospective analysis of 144 cases revealed that bone pain was the most common reported symptom, followed by height loss in more than 50% of patients [15]. Pathological fractures of both thoracic and lumbar vertebrae, ribs, femurs, and the pelvis were the major cause of morbidity associated with TIO [13].

The typical biochemical pattern includes low serum phosphate, normal to low calcium, normal parathyroid hormone (PTH), low or inappropriately normal 1,25-dihydroxy vitamin D, normal 25-hydroxyvitamin D, elevated alkaline phosphatase, and elevated fibroblast growth factor 23 (FGF-23) concentrations in the serum. Increased phosphate excretion in urine is demonstrated by a low tubular reabsorption of phosphate [16]. The usual presentation is generally through investigation for fragility fractures, bone pain, muscle weakness, and height loss [15].

The principal pathogenic mechanism of TIO is over-production of fibroblast growth factor 23 (FGF23), a phosphatonin that causes renal phosphate wasting, secondary to it is role in regulating renal inorganic phosphate reabsorption and modifying 25-hydroxyvitamin D 1α-hydroxylase activity [17]. We did not have access to measurement of serum FGF-23.

The differential diagnosis of TIO includes genetic causes of hypophosphatemic osteomalacia, in particular, X-linked hypophosphatemic rickets (XLH), autosomal dominant hypophosphatemic rickets (ADHR), and autosomal recessive hypophosphatemic rickets (ARHR) [18]. These aforementioned conditions may manifest with a similar phenotype to TIO, but usually a positive family history is elicited, with early onset gait and skeletal deformities. Both TIO and these inherited forms of hypophosphatemic rickets are associated with elevated FGF23. The positive family history may corroborate a genetic cause for hypophosphatemia, which is further proven by genetic testing, including PHEX mutation for XLH, mutations in the FGF23 gene causing ADHR (including *R176Q*, *R176W*, *R179Q*, *R179W*), and mutations for autosomal recessive hypophosphatemic rickets (ARHR), including inactivating mutations in the *DMP1* gene, *ENPP1* gene, and mutations in the *FAM20C* gene encoding a protein kinase [19–21]. FGF-23 is presumed to reduce the expression of both 1-α-hydroxylase enzyme and two phosphate channels namely [Na-Pi 2a) and (Na-Pi 2c)], leading to phosphate wasting in the intestine and kidneys [22-25].

A logical stepwise process has been proposed to locate the offending tumor, by combining functional and anatomical tests, using PET/CT scan, CT scan, and MRI [14]. These tumors sometimes express somatostatin receptors [26, 27], which facilitates location, using somatostatin-receptor (SSTR) imaging, but they lack specificity, necessitating a pathological diagnosis [28] Following functional imaging, anatomical imaging is necessary to confirm a pathological lesion using ultrasound, CT, and MRI, depending on the location [13]. Despite all the advances in the investigations for diagnosis, there is a proportion of patients who fail to demonstrate a positive result. Imaging may be repeated every 1–2 years in the hope that the tumor may declare itself [13].

Medical therapy is indicated during workup of these patients, when tumors cannot be located or a complete excision cannot be performed [29]. The basic regimen for TIO includes phosphate and active vitamin D (calcitriol and alfacalcidol) supplements [30] Side effects include nephrolithiasis, nephrocalcinosis, impaired renal function, and either secondary or tertiary hyperparathyroidism [29], necessitating close surveillance. Somatostatin receptor analogues have been used with some success [29, 31–33]. Inconsistent results are reported regarding the efficacy of somatostatin receptor-based therapy with octreotide in this setting [31–33]. FGF-23 antibodies, for example, burosumab, a fully human IgG1 monoclonal antibody directed at fibroblast growth factor 23, is now licensed to be used in the treatment of TIO [33].

## Conclusions

TIO remains a rare, but potentially treatable, paraneoplastic syndrome, requiring a high index of suspicion. Careful attention in assessing patients for osteoporosis who also have a low serum phosphate, should alert the clinician to either a hereditary form of hypophosphatemia or an oncogenic condition, such as tumor-induced osteomalacia.

## Data Availability

All laboratory data are available through the repository held by the National Health Laboratory Services (NHLS), South Africa and radiology is available from the Department of Radiology, Groote Schuur Hospital, Cape Town.

## Abbreviations

TIO: Tumor-induced osteomalacia
FGF-23: Fibroblast growth factor 23
DEXA: Dual energy x-ray absorptiometry
SD: Standard deviations
Ga 68-DOTANOC PET/CT: Gallium 68 (68Ga) 1,4,7,10-tetraazacyclododecane-1,4,7,10-tetraacetic acid (DOTA)–octreotate (DOTATATE, GaTate) positron emission tomography/computed tomography
MRI: Magnetic resonance imaging
XLH: X-linked hypophosphatemic rickets
ADHR: Autosomal dominant hypophosphatemic rickets
ARHR: Autosomal recessive hypophosphatemic rickets
PHEX: Phosphate regulating gene with homologies to endopeptidases on the X-chromosome
DMP1: Dentin matrix acidic phosphoprotein 1
ENPP1: Ectonucleotide pyrophosphatase/phosphodiesterase-1
FAM20C: Family with sequence similarity 20, member C
PET/CT: Positron emission tomography/computed tomography
CT: Computed tomography
SSTR: Somatostatin receptor
IgG1: Immunoglobulin G1
